# Treatment of Obstructive Sleep Apnea With Mandibular Advancement Device (MAD) in a 58‐year‐old Female Patient With Psychiatric Comorbidity: Case Report With 6‐Month Follow‐up

**DOI:** 10.1155/crid/4064872

**Published:** 2026-06-25

**Authors:** Angela Mirea Bellocchio, Ludovica Ciraolo, Maria Fazio, Riccardo Nucera

**Affiliations:** ^1^ Department of Biomedical and Dental Sciences and Morpho-Functional Imaging, Section of Orthodontics, School of Dentistry, University of Messina, Messina, Italy, unime.it; ^2^ Department of Mathematical and Computer Sciences, Physical Sciences, and Earth Sciences, University of Messina, Messina, Italy, unime.it

**Keywords:** case report, CPAP intolerance, depression, mandibular advancement device, obstructive sleep apnea

## Abstract

**Background:**

Obstructive sleep apnea (OSA) and major depressive disorder frequently coexist, creating a complex bidirectional relationship that influences diagnosis and treatment adherence. Continuous positive airway pressure (CPAP) remains the gold standard for OSA treatment; however, poor tolerance or refusal is common, particularly among patients with psychiatric comorbidities.

**Case Report:**

A case of a 58‐year‐old female patient with moderate OSA and major depressive disorder who declined CPAP therapy due to psychological discomfort is presented. Following multidisciplinary evaluation, a mandibular advancement device (MAD) was implemented as an alternative treatment. Over a 6‐month follow‐up, significant improvements were observed in OSA severity and depressive symptoms, including increased oxygen saturation, reduced apneic events, and enhanced mood, despite stable pharmacological treatment.

**Conclusions:**

This case demonstrates the clinical effectiveness of MADs in managing OSA in patients intolerant to CPAP, especially those with psychiatric comorbidities. The importance of interdisciplinary collaboration in the management of complex cases is also emphasized.

## 1. Introduction

Obstructive sleep apnea (OSA) and depression are two chronic conditions frequently associated with a complex, bidirectional relationship. OSA is characterized by recurrent episodes of partial or complete obstruction of the upper airway during sleep, leading to intermittent hypoxemia, sleep fragmentation, and persistent activation of the sympathetic nervous system. These physiological alterations promote systemic inflammatory responses and may interfere with neurotransmitter pathways involved in mood regulation, including serotonin, dopamine, and norepinephrine [1, 2].

As a consequence, OSA has been associated not only with excessive daytime sleepiness, cardiovascular and metabolic complications, but also with neuropsychiatric manifestations such as depressive symptoms, cognitive impairment, and reduced quality of life.

Depression itself is a multifactorial disorder involving dysregulation of the hypothalamic–pituitary–adrenal (HPA) axis, increased oxidative stress, and chronic low‐grade inflammation [3, 4].

The coexistence of OSA may amplify these pathophysiological mechanisms, generating a vicious cycle in which sleep‐disordered breathing worsens mood disturbances, whereas depressive symptoms negatively affect sleep quality and adherence to treatment. Recent evidence indicates that untreated OSA may increase the risk of developing depressive symptoms and may also attenuate the effectiveness of pharmacological and psychotherapeutic interventions commonly used in the management of depression [5, 6].

OSA affects a significant proportion of the adult population, with prevalence estimates exceeding 10%. In addition to systemic and neurochemical mechanisms, growing attention has been directed toward the role of craniofacial and dento‐palatal morphology in the pathogenesis and severity of the disease [7]. Several craniofacial characteristics—including reduced mandibular length, mandibular retrusion, alterations of the cranial base, and narrow or high‐arched palatal morphology—may contribute to reduced upper airway dimensions and increase the susceptibility to airway collapse during sleep. In particular, a decreased maxillary arch width and altered palatal morphology have been associated with higher apnea–hypopnea index (AHI) and oxygen desaturation index (ODI) values. These findings highlight the relevance of orthodontic and craniofacial assessment in the multidisciplinary evaluation of patients with OSA, suggesting that orthodontists may contribute not only to treatment planning but also to the early identification of individuals at increased risk and to timely referral within an integrated clinical pathway [8, 9].

The first‐line treatment for moderate‐to‐severe OSA is continuous positive airway pressure (CPAP), which prevents pharyngeal collapse and improves respiratory parameters [10].

However, adherence to CPAP therapy remains a major challenge in clinical practice. Many patients experience discomfort, a perception of invasiveness, or difficulties tolerating the device during nighttime use, factors that may compromise long‐term adherence. These challenges may be even more relevant in patients with psychiatric comorbidities [11], including depressive disorders [12].

In this context, alternative therapeutic options that are better tolerated may play an important role. Mandibular advancement devices (MADs) represent a noninvasive treatment modality designed to advance the mandible during sleep, thereby increasing upper airway volume and reducing the likelihood of pharyngeal collapse. Traditionally indicated for patients with mild‐to‐moderate OSA or for individuals unable to tolerate CPAP therapy, MAD treatment has gained increasing clinical relevance with the development of customized appliances fabricated through conventional impressions or digital scanning technologies, which improve comfort, precision, and therapeutic effectiveness [13, 14].

In this context, the present case report describes the management of OSA using a customized MAD in a 58‐year‐old female patient with psychiatric comorbidity, highlighting the multidisciplinary diagnostic pathway and the clinical outcomes observed during a 6‐month follow‐up period.

This case report was prepared in accordance with the CARE (CAse REport) guidelines.

## 2. Case Presentation

A 58‐year‐old postmenopausal female patient presented for evaluation and management of OSA. She was under treatment at the Neurology Outpatient Clinic, University Hospital of Messina, for major depressive disorder (MDD) and generalized anxiety disorder, and was receiving venlafaxine 75 mg/day, along with medications for comorbid conditions. Her medical history was notable for daytime fatigue, carbohydrate cravings, anhedonia, low libido, and lack of motivation. A diagnosis of moderate OSA was confirmed via overnight polysomnography (21 May 2024), revealing an AHI of 20.5 events/hour.

### 2.1. Pretreatment Clinical and Diagnostic Assessment

#### 2.1.1. Clinical Examination

**Figure 1:**
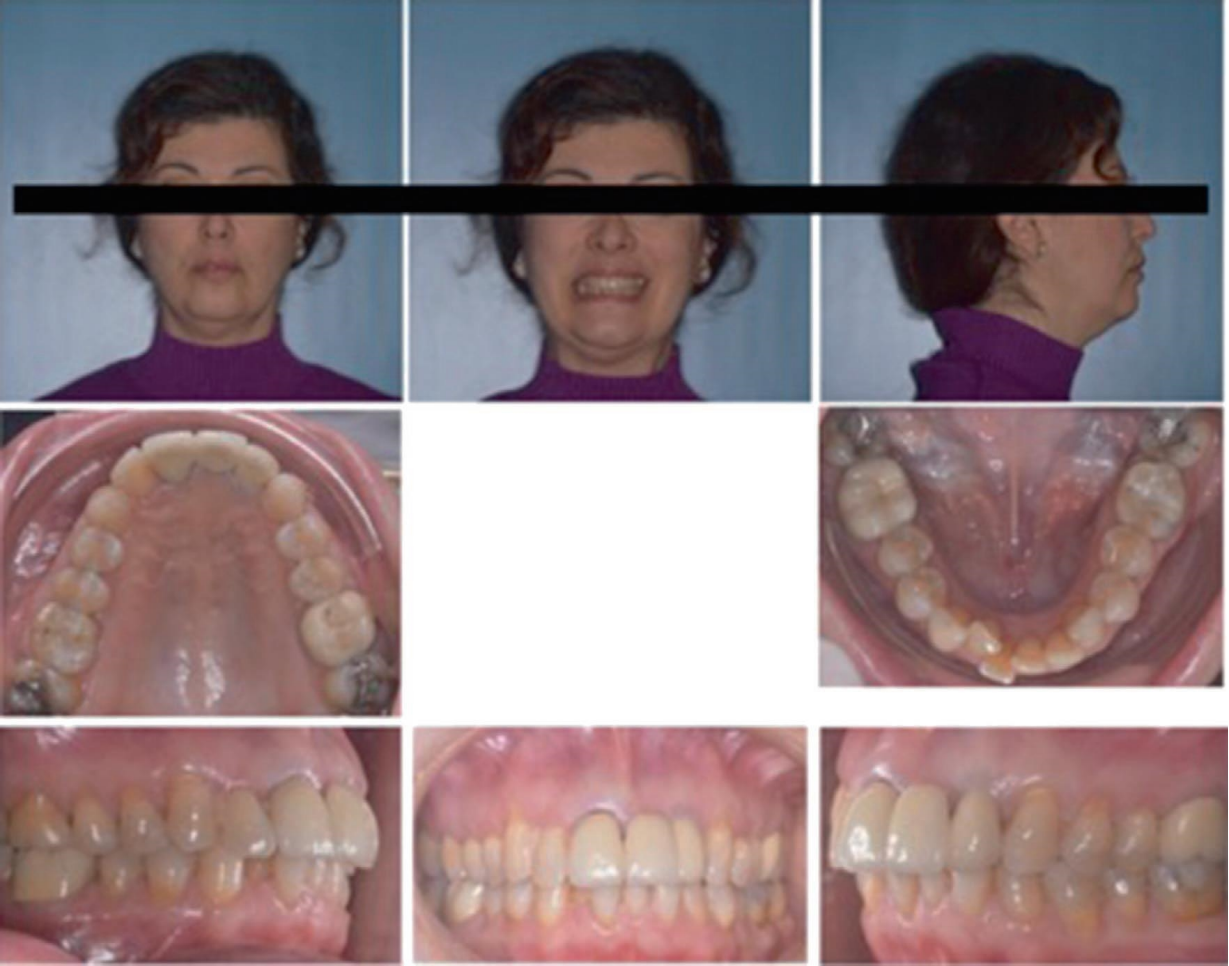
Intraoral and extraoral photographs of the patient. The images show the overall clinical presentation, including external facial features and the oral cavity.

The patient reported habitual snoring, morning coughing, and persistent dysphonia, accompanied by symptoms of gastroesophageal reflux disease (GERD). Physical examination revealed a Mallampati score of 4/4, oral asymmetry, and a neck circumference of 33 cm—indicators suggestive of an elevated risk for upper airway obstruction. (Figure 1).

**Table 1:** Comparison of polysomnographic and clinical parameters before and after treatment.

Parameter	Pretreatment (T0)	Posttreatment (T1)	Change
Apnea‐hypopnea index (AHI)	20.5	11.3	↓ 9.2
Mean oxygen saturation (%)	93.4%	94%	≈
T90 (time with SpO₂<90*%*) (%)	8.2%	0.7%	↓ 7.5%
Minimum SpO_2_ (%)	81%	84%	↑ 3%
Mean heart rate (bpm)	81.2	78	↓ 3.2 bpm
Snoring index (%)	2.5%	0.1%	↓ 2.4%
ODI (events/hour)	38.6	5.4	↓ 86%
Epworth Sleepiness Scale (ESS)	5	2	↓ 3
Beck depression inventory (BDI)	11	4	↓ 7
Hamilton anxiety rating scale (HAM‐A)	14	9	↓ 5

#### 2.1.2. Polysomnography (Baseline, T0)

The polysomnography is decribed as follows:

• AHI: 20.5 events/hour (moderate OSA)
• Mean oxygen saturation: 93.4%
• T90 (percentage of sleep time with SpO_2_ < 90*%*): 8.2%
• Mean heart rate: 81.2 bpm
• Minimum SpO_2_: 81%
• ODI: 38.6 events/hour

#### 2.1.3. Psychological Assessment (T0)

The psychological assessments are listed as follows:

• Epworth Sleepiness Scale (ESS): 5 (mild daytime sleepiness)
• Beck Depression Inventory (BDI): 11 (mild depressive symptoms)
• Hamilton Anxiety Rating Scale (HAM‐A): 14 (moderate anxiety)

#### 2.1.4. Laboratory Findings

Routine hematological screening was unremarkable except for elevated serum ferritin (216 ng/mL), potentially reflecting subclinical inflammation or iron overload.

#### 2.1.5. Therapeutic Strategy

Due to intolerance to CPAP, a MAD was selected as the primary noninvasive intervention. MADs function by advancing the mandible to increase upper airway patency and reduce collapsibility during sleep, thereby mitigating apneic episodes, enhancing sleep quality, and potentially improving mood and energy.

#### 2.1.6. Dental and Gnathological Assessment

The dental and gnathological assessment are detailed as follows:

• Dentition: adequate for device retention.
• Airway anatomy: no macroglossia or tonsillar hypertrophy.
• Temporomandibular joint (TMJ) function: normal, without dysfunction or movement limitation.
• Mandibular protrusion: achieved approximately 70% of maximal protrusion, measured using the George Gauge.

#### 2.1.7. Radiographic Imaging

**Figure 2:**
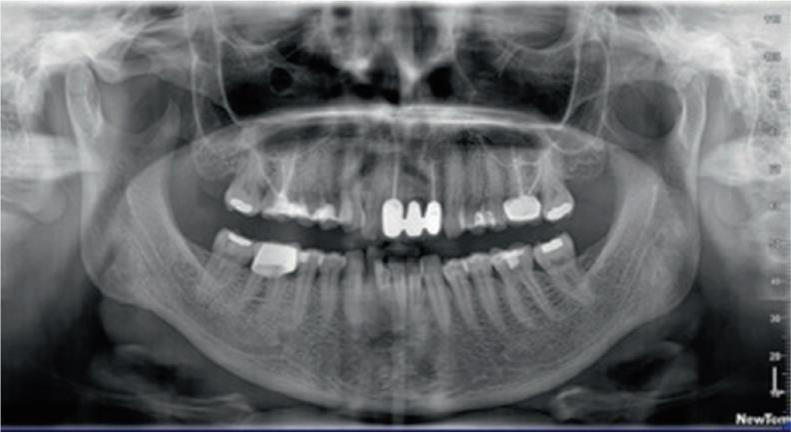
Orthopantomogram (OPT) radiograph showing the panoramic view of the patient′s dental structures.

The radiographic imaging is described as follows:

• Orthopantomogram (OPT): normal dentition and condylar morphology. (Figure 2).

#### 2.1.8. MAD Fabrication and Delivery Protocol

Following thorough clinical and radiographic evaluation—including panoramic radiography and gnathological examination—the patient′s dental condition was deemed suitable for MAD therapy. No periodontal issues, extensive restorations, or TMJ contraindications were identified.

**Figure 3:**
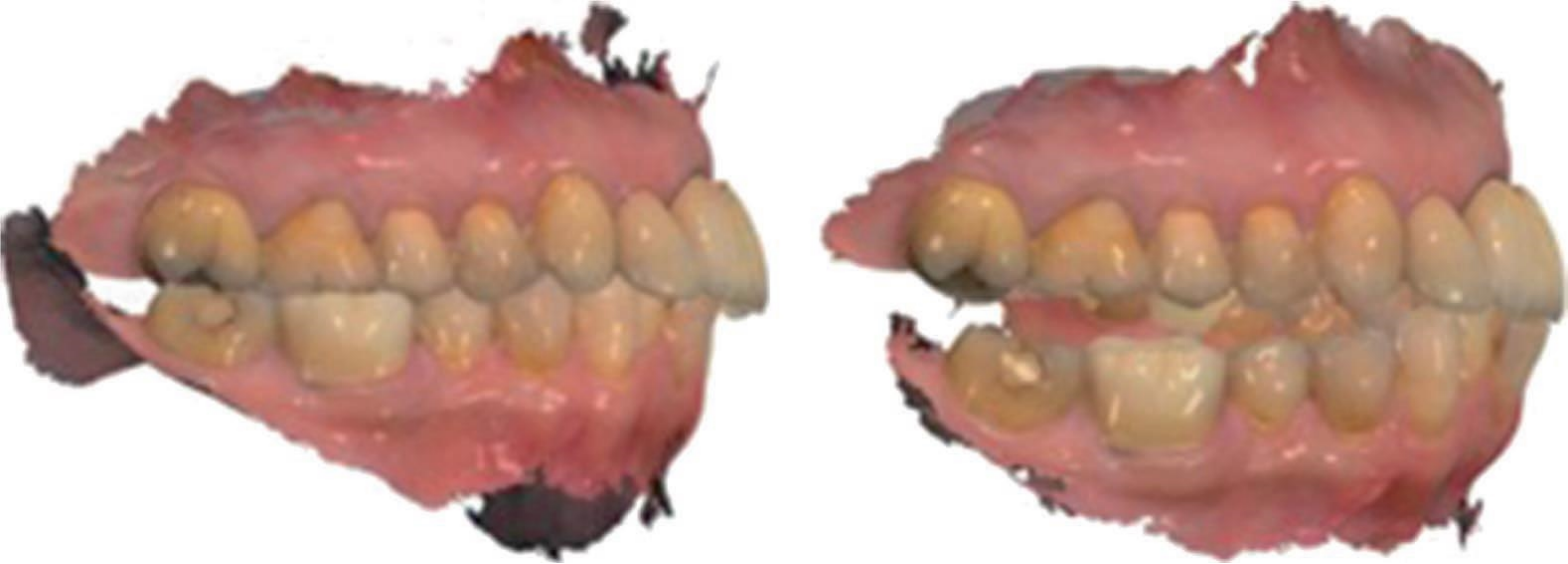
Intraoral scans (STL) performed with an intraoral scanner, both with and without the mandibular advancement. The scans illustrate the changes in dental arch configuration and mandibular positioning when the device is in place compared with the natural state.

Digital impressions of both arches were obtained using a high‐resolution intraoral scanner (3Shape TRIOS 4, 3Shape, Copenhagen, Denmark), ensuring precise virtual models and eliminating the distortions typical of conventional impressions. Mandibular protrusion was registered at approximately 70% of the patient′s maximum comfortable advancement via the George Gauge (Great Lakes Dental Technologies, New York, United States), optimizing therapeutic effect while maintaining muscular comfort without pain or myofascial strain. (Figure 3).

Working models were 3D‐printed with a desktop stereolithographic printer (Elegoo Saturn 2, Elegoo Inc., Shenzhen, China) using biocompatible resin (Class I, ISO 10993 certified). Upper and lower thermoplastic splints were thermoformed from medical‐grade polyurethane sheets (Erkodur, 1.5 mm thickness) over the models using a pressure molding system (Erkopress 300 Tp, Erkodent).

Before finalizing the advancement mechanism, the splints were delivered for a 48‐h acclimatization period. During this phase, the patient received instructions on oral hygiene and device care, including mechanical cleaning and daily disinfection with a chlorhexidine‐free solution to minimize mucosal irritation and promote adherence.

**Figure 4:**
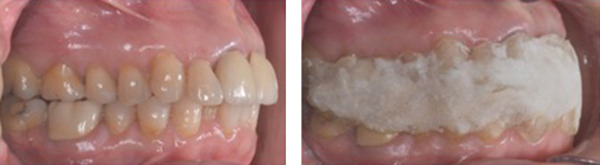
Intraoral lateral photographs of the patient, showing the dental arch both with and without the mandibular advancement device used for the treatment of obstructive sleep apnea.

At follow‐up after 72 h, the patient reported no discomfort, pain, or speech disturbances. Consequently, the splints were secured in the predetermined protrusive position with a biocompatible light‐curing resin (ProBase Cold, Ivoclar Vivadent), ensuring stable interarch fixation while preserving physiological vertical dimension (Figure 4).

To ensure physiological condylar seating and neuromuscular balance, the interarch connector was polymerized intraorally using cold‐cure PMMA (ProBase Cold, Ivoclar Vivadent), rather than designing the coupling digitally from the protrusive‐registered scan. This approach enabled real‐time verification of mandibular comfort and symmetrical loading during advancement. The digital scans remain essential for ensuring accurate splint fit and enabling future titration without remaking the device.

ProBase Cold is a medical‐grade PMMA compliant with ISO 20795‐1 standards. It exhibits low residual monomer (< 1.5 wt%), homogeneous polymerization and low porosity under pressure‐curing, resistance to creep and fatigue under cyclic occlusal forces, and compatibility with commonly used alkaline peroxide‐based cleansers.

**Figure 5:**
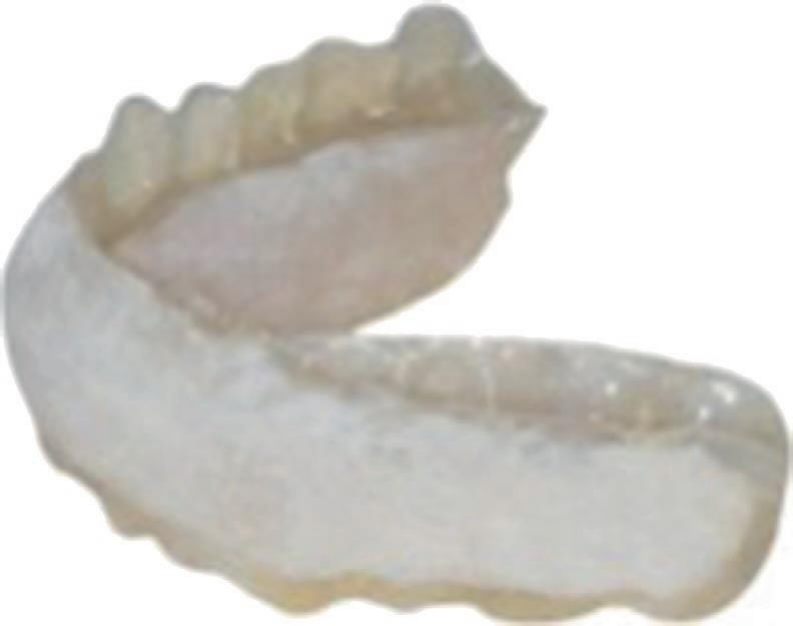
Finished custom mandibular advancement device (MAD) made of medical‐grade polyurethane (Erkodur). The interarch connector, fabricated intraorally with cold‐cure PMMA (ProBase Cold, Ivoclar Vivadent), has been refined and polished following the standardized laboratory finishing protocol.

To minimize plaque accumulation and mucosal irritation, the connector was shaped with a continuously convex profile, intentionally avoiding any undercuts or rough transition zones. After polymerization, the surface was finished using a standardized multistep protocol: trimming with tungsten carbide burs, refining with medium–fine grit silicone polishers, and final polishing with pumice and PMMA high‐gloss paste. The device was then refined in the laboratory. (Figure 5).

Surface roughness was measured using a contact profilometer (Mitutoyo Surftest SJ‐210). The mean values obtained were *R* *a* = 0.12–0.22 * μ*m and *R* *z* = 0.8–1.5 * μ*m, which are below the 0.2 *μ*m threshold associated with reduced bacterial adhesion.

To evaluate cleanability and durability, the device underwent brushing simulation (10,000 cycles), immersion in alkaline peroxide cleanser (30 cycles), thermal cycling (5–55°C, 500 cycles), and water storage (37°C, 7 days). Digital superimposition (3Shape Ortho Analyzer) confirmed no deformation > 0.1 mm; surface roughness remained stable (*Δ*Ra < 0.03 *μ*m), and no microfractures or delamination were observed.

All procedures complied with ISO 22674 and ISO 20795‐1 standards for intraoral medical devices. Informed consent was obtained after detailed counseling regarding the mechanism, expected benefits, and potential risks of MAD therapy.

### 2.2. Clinical Follow‐Up and Outcomes

#### 2.2.1. 1‐Month Follow‐Up

The patient reported marked improvements in:

• Snoring: significant reduction in frequency and intensity.
• Sleep quality: enhanced continuity, fewer awakenings, and increased daytime alertness.
• Mood and psychosocial function: notable stabilization despite unchanged pharmacotherapy.
• BDI score: decreased from 11 to 4, indicating remission of mild depressive symptoms.
• TMJ and oral function: no discomfort, preserved mandibular mobility, and no adverse periodontal effects.

### 2.3. 6‐Month Follow‐Up—Polysomnographic and Psychological Reassessment

#### 2.3.1. Polysomnography (T1)

The polysomnogradphy (T1) is described as follows:

• AHI: reduced to 11.3 events/hour (mild OSA).
• Mean oxygen saturation: stable at 94%.
• T90 (SpO_2_ < 90*%*): decreased to 0.7%.
• Mean heart rate: 78 bpm.
• Minimum SpO_2_: 84%.
• ODI: 5.4 events/hour.

Archived PSG tracings confirmed a substantial reduction in apneic/hypopneic events, improved sleep architecture, and enhanced oxygenation without adverse effects or device intolerance.

#### 2.3.2. Psychological Assessment (T1)

The psychological assessment (T1) is described as follows:

• ESS: two (minimal daytime sleepiness).
• BDI: four (improved mild depressive symptoms).
• HAM‐A: nine (minimal anxiety).

The following summarizes the clinical, polysomnographic, and psychological parameters recorded before and after MAD therapy:

## 3. Results

After 6 months of treatment with the MAD, the patient demonstrated notable improvements in respiratory, cardiovascular, and psychological parameters (Table 1).

### 3.1. Respiratory Outcomes

The AHI decreased from 20.5 events/hour at baseline (T0; moderate OSA) to 11.3 events/hour posttreatment (T1; mild OSA), indicating a clinically meaningful reduction in apneic events. Oxygenation also improved: Mean oxygen saturation increased slightly from 93.4% to 94%, minimum saturation rose from 81% to 84%, and the percentage of sleep time spent below 90% saturation (T90) decreased from 8.2% to 0.7%, reflecting better nocturnal oxygenation. A marked improvement was also observed in the ODI, which decreased from 38.6 events/hour at baseline to 5.4 events/hour after treatment, indicating a substantial reduction in the frequency of nocturnal oxygen desaturation events and improved respiratory stability during sleep. Snoring intensity, as measured by the snoring index, was almost completely eliminated, dropping from 2.5% to 0.1%.

### 3.2. Cardiovascular Outcomes

The patient′s mean nocturnal heart rate decreased from 81.2 bpm at baseline to 78 bpm after treatment, suggesting a modest improvement in cardiovascular regulation during sleep. This reduction may reflect decreased sympathetic nervous system activation associated with the reduction of apneic events and intermittent hypoxia. The concomitant decrease in ODI further supports an improvement in nocturnal oxygenation and autonomic balance, factors that are closely linked to cardiovascular stress in patients with OSA.

### 3.3. Psychological Outcomes

Improvements in sleepiness, mood, and anxiety were observed in parallel. Daytime sleepiness, assessed by the ESS, decreased from 5 to 2. Depressive symptoms, measured with the BDI, improved substantially from 11 to 4, whereas anxiety levels, assessed using the HAM‐A, decreased from 14 to 9.

Overall, MAD therapy was associated with clinically relevant improvements in both objective respiratory parameters and subjective psychological measures, demonstrating its effectiveness in this patient over a 6‐month follow‐up period.

## 4. Discussion

This case demonstrates the efficacy of a MAD as an alternative therapy for OSA in a patient with comorbid MDD and intolerance to CPAP. Clinical improvements observed in both respiratory function and psychological symptoms underscore the importance of a multidisciplinary and personalized approach in patients with complex comorbid conditions.

OSA is associated with systemic inflammatory processes and neurochemical dysregulation that contribute to the onset and persistence of depression [15].

Several neurophysiological mechanisms have been implicated in this bidirectional relationship. Chronic intermittent hypoxia—a hallmark of OSA—induces oxidative stress and promotes the release of proinflammatory cytokines such as interleukin‐6 (IL‐6) and tumor necrosis factor‐alpha (TNF‐*α*), which are known to affect central nervous system function and contribute to mood disturbances. Additionally, repeated nocturnal arousals activate the HPA axis, resulting in sustained elevations of cortisol levels that disrupt circadian rhythms and impair neuroplasticity, particularly in limbic structures involved in emotional regulation, such as the amygdala and hippocampus. These pathophysiological changes may lead to alterations in monoaminergic transmission and sleep architecture, both of which are critically involved in the pathogenesis of depressive and anxiety disorders [16, 17].

The bidirectional relationship between these disorders may impair adherence to standard therapies such as CPAP, especially in the presence of psychiatric comorbidities [18].

The favorable response to MAD therapy, characterized by a reduction in apneic events and improved nocturnal oxygenation, was accompanied by significant improvement in depressive and anxiety symptoms despite stable pharmacological treatment. Although the MAD did not completely normalize the AHI, the observed reduction from moderate to mild OSA is considered clinically meaningful and consistent with the expected therapeutic effect of oral appliance therapy in patients intolerant to CPAP. The absence of mandibular or occlusal adverse effects in this case supports the short‐term safety and tolerability of MAD therapy. Nonetheless, the potential long‐term impact on TMJ function and occlusal stability remains debated. Some studies have reported risks of joint discomfort, dysfunction, or occlusal changes with prolonged use—particularly in cases of significant mandibular advancement—raising concerns about excessive load on TMJ structures [19].

Some studies have reported an increased risk of joint discomfort, dysfunction, or occlusal changes following prolonged use, whereas others suggest an acceptable safety profile when patients are closely monitored [20, 21]. Conversely, other longitudinal studies, including those using MRI, have shown no worsening of temporomandibular disorders and, in some cases, even clinical improvement in joint sounds and muscle pain [22, 23]. These findings highlight the importance of regular clinical follow‐up to ensure functional adaptation and prevent complications. From a clinical perspective, this case highlights several key points: (1) MADs represent a valid therapeutic alternative for patients intolerant to CPAP, particularly in the presence of psychiatric comorbidities; (2) therapeutic efficacy should be assessed not only in terms of respiratory indices but also considering psychological outcomes; and (3) rigorous monitoring of TMJ function and occlusal stability is essential to balance therapeutic benefits with safety. The decision to finalize mandibular advancement intraorally, rather than relying exclusively on digital superimposition, allowed direct control of condylar positioning and neuromuscular equilibrium during functional protrusion. This clinician‐guided adjustment may represent an important step in optimizing airway opening and improving therapeutic effectiveness. Although digital scans ensure splint precision, small variations in the spatial relationship of the arches during advancement may lead to suboptimal airway opening if corrected only virtually. Intraoral PMMA bonding enables clinician‐guided titration of the effective therapeutic position. This approach is consistent with the functional philosophy of mandibular advancement therapy and remains reversible, as the PMMA connector can be replaced if adaptation changes occur over time. This case is clinically relevant because it highlights the potential role of MADs not only as a mechanical treatment for OSA but also as a therapeutic option that may indirectly contribute to psychological improvement in patients with psychiatric comorbidities who are unable to tolerate CPAP therapy.

Future research should focus on elucidating the neurobiological mechanisms underlying mood improvement associated with OSA therapy, including the modulation of neuroinflammatory markers, HPA axis activity, and sleep architecture. Randomized controlled trials comparing MADs and CPAP in patients with psychiatric comorbidities are warranted to define optimal patient selection criteria and establish evidence‐based, multidisciplinary treatment algorithms.

A limitation of this report is that it describes a single clinical case, which limits the generalizability of the findings. Nevertheless, the detailed clinical, polysomnographic, and psychological follow‐up provides useful insights into the multidisciplinary management of patients with OSA and psychiatric comorbidities.

## 5. Conclusions

In this case report, the MAD proved to be an effective alternative to CPAP, leading to significant improvements in respiratory parameters and sleep quality in a patient with OSA and psychiatric comorbidity.

Treatment was also associated with psychological benefits, including a reduction in depressive and anxiety symptoms, despite stable pharmacological therapy.

From the patient′s perspective, MAD therapy was well tolerated and associated with improved quality of life and treatment adherence.

Short‐term use of the MAD was safe and well tolerated; nevertheless, regular monitoring of TMJ function and occlusal stability remains essential to prevent potential adverse effects.

Future controlled studies are warranted to elucidate the neurobiological mechanisms underlying mood improvement and to establish standardized, evidence‐based protocols for the management of patients with psychiatric comorbidities.

## Author Contributions

Conceptualization: Angela Mirea Bellocchio and Riccardo Nucera. Methodology: Angela Mirea Bellocchio and Riccardo Nucera. Clinical investigation: Angela Mirea Bellocchio and Ludovica Ciraolo. Data collection: Angela Mirea Bellocchio and Ludovica Ciraolo. Data analysis and interpretation: Angela Mirea Bellocchio and Maria Fazio. Writing—original draft preparation: Angela Mirea Bellocchio. Writing—review and editing: Angela Mirea Bellocchio, Ludovica Ciraolo, Maria Fazio, and Riccardo Nucera. Supervision: Riccardo Nucera.

## Funding

Corresponding author is a researcher supported by the European Union (NextGeneration EU), through the MUR‐PNRR SAMOTHRACE project (ECS000022). The support covers general doctoral training and research activities. Open access publishing facilitated by Universita degli Studi di Messina, as part of the Wiley ‐ CRUI‐CARE agreement.

## Disclosure

All authors have read and approved the final version of the manuscript. The corresponding author had full access to all of the data in this study and takes complete responsibility for the integrity of the data and the accuracy of the data analysis.

## Ethics Statement

Ethical approval was not required for this study in accordance with institutional guidelines, as it represents a single anonymized case report.

## Consent

Written informed consent was obtained from the patient for publication of this case report and the accompanying images.

## Conflicts of Interest

The authors declare no conflicts of interest.

## Data Availability

The raw data supporting the findings of this study are available from the corresponding author upon reasonable request.
